# Reattachment of Fractured Tooth Fragment with Fiber Post: A Case Series with 1-Year Followup

**DOI:** 10.1155/2014/376267

**Published:** 2014-11-03

**Authors:** C. M. Sapna, R. Priya, N. B. Sreedevi, Rakesh R. Rajan, Renjith Kumar

**Affiliations:** Department of Conservative Dentistry & Endodontics, Amrita School of Dentistry, AIMS Ponekkara P. O., Kochi 682041, India

## Abstract

Coronal fractures of the anterior teeth are common sequelae of dental trauma. In case of complex fractures, where the fractured segment is available and there is close approximation of the segment to the remaining tooth, root canal treatment followed by reattachment of the fractured segment with fiber post reinforcement is a feasible option. The procedure is simple and economic and needs less chair-side time as compared to many conventional methods. In addition, the procedure provides good and long-lasting esthetics, because the original morphology, color, and surface texture are maintained. This paper reports three cases of complex coronal tooth fracture successfully managed using tooth fragment reattachment.

## 1. Introduction

Complicated crown fractures involving the enamel, dentin, and pulp constitute a major share of all dental injuries and are most common in maxillary central incisors [1, 2]. A fractured anterior tooth requires immediate clinical attention and, if untreated, can cause damage to dentition and even have a psychological impact on the patient [1].

Management of complicated crown fractures is a multifactorial process influenced by the extent and pattern of fracture (biological width violation, endodontic involvement, alveolar bone fracture), restorability of fractured tooth (associated root fracture), secondary injuries (soft tissue status), presence/absence of fractured tooth fragment and its condition for use (fit between fragment and the remaining tooth structure), occlusion, esthetics, finances, and prognosis [3]. In case of complicated fractures where the fractured segments are closely approximating, root canal treatment (RCT) followed by reattachment of the fractured segment with fiber post reinforcement is a feasible option [3]. It has been suggested that fiber post luted with resin cement increases the retention of the segment and also provides a monoblock effect [4].

This paper reports three cases of complicated coronal tooth fracture successfully managed by tooth fragment reattachment.

## 2. Case Reports

### 2.1. Case  1

**Figure 1:**
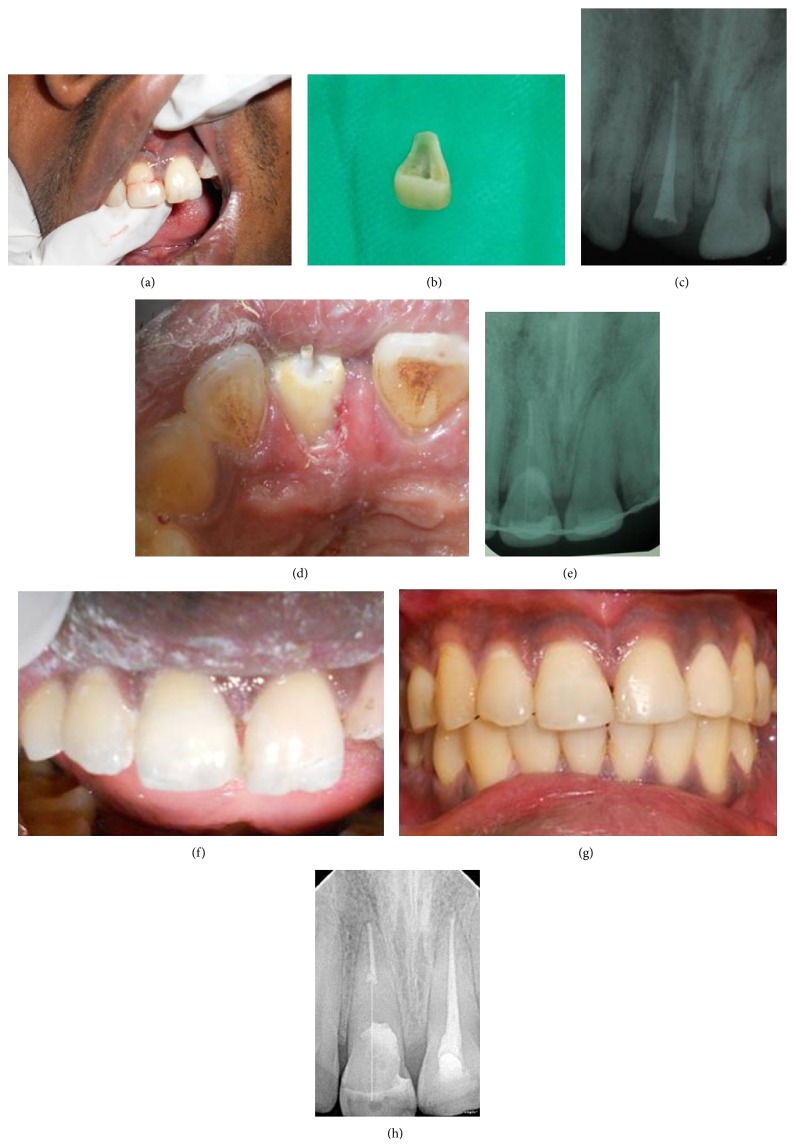
(a) Preoperative view. (b) Fractured segment. (c) Postobturation 11. (d) Post luted on 11. (e) Postoperative radiograph. (f) Postoperative view. (g) 1-year follow-up view. (h) 1-year follow-up radiograph.

A 27-year-old male injured in a road traffic accident (RTA) was referred to the Department of Conservative Dentistry and Endodontics, Amrita School of Dentistry. Clinical and radiographic examination revealed a complicated oblique crown fracture on 11 that extended subgingivally in the mesiopalatal aspect. The fractured segment was held in place by the gingival attachment (Figure 1(a)). Ellis class III fracture of 22 was also noted but the fractured segment was missing. Periapical radiographs revealed an intact periodontal ligament space, complete root formation, and no root fracture in relation to both teeth. Medical history was noncontributory.

It was planned to perform single visit root canal treatment (RCT) on 11 followed by reattachment with fiber post reinforcement. 22 was also planned for RCT and post-core restoration.

Local anesthesia was administered (1.0 cc of lidocaine 2% with 1 : 80,000 epinephrine) and the fractured segment in relation to 11 was atraumatically removed (Figure 1(b)). It was then cleaned with 2% chlorhexidine solution and stored in isotonic saline solution. RCT was completed on 11 (Figure 1(c)) and post space was prepared using GG drills and Peeso reamers. Esthetic post of diameter 1.1 mm (Angelus, REFORPOST, Londrina, Brazil) was selected. The prepared post space was etched for 15 seconds using 37% phosphoric acid (DPI Tooth conditioner gel, Dental Products of India, Mumbai, India). It was then rinsed thoroughly with water and excess water was removed with a cotton pellet. Next the adhesive (Prime & Bond NT, Nanotechnology Dental adhesive, Dentsply, St. Paul, MN, USA) was applied on the etched surface as well as the post. The adhesive was air-thinned and light-cured for 10 seconds. The post was then luted with resin cement (Multilink, Ivoclar, Vivadent) with 2 mm of its coronal portion extending into the chamber (Figure 1(d)). Tooth fragment was reattached using resin cement (Figures 1(e) and 1(f)), and a physiological splint using ligature wire and composite resin (Ceram-X Mono^+^ Nanoceramic Restorative, Denstply, Konstanz, Germany) was provided for 2 weeks. The RCT for 22 and subsequently 21 was performed. The patient was kept on periodic review and it was observed that both endodontic and restorative treatments remained clinically acceptable through each visit. The clinical and radiographic pictures after 1 year revealed favorable healing (Figures 1(g) and 1(h)).

### 2.2. Case  2

**Figure 2:**
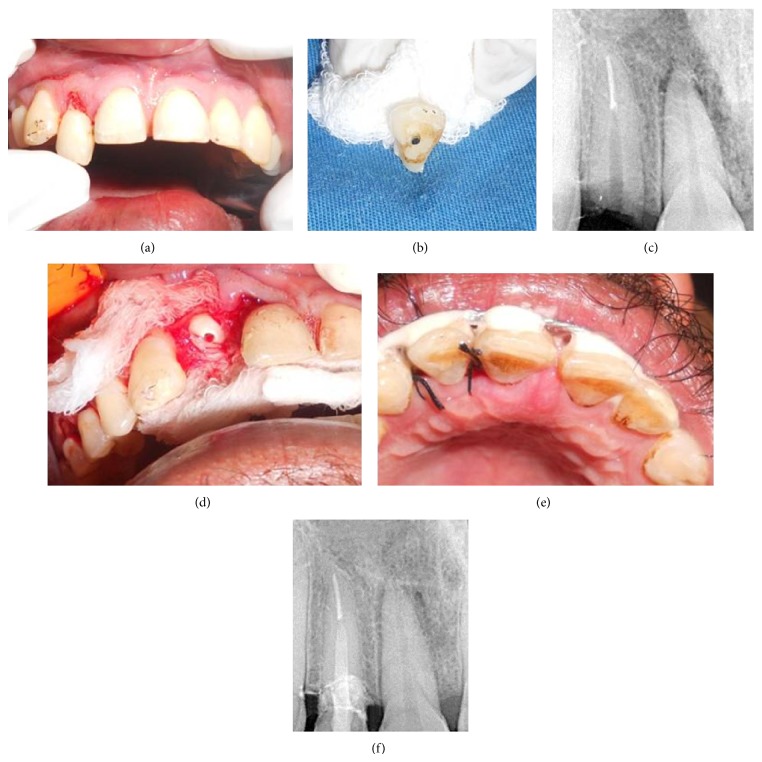
(a) Preoperative view. (b) Fractured segment. (c) Radiograph of prepared post space. (d) Flap elevated to expose margins of fracture. (e) Postoperative view. (f) 1-year follow-up radiograph.

A similar case of a 28-year-old male presented with Ellis class III fracture of the right maxillary lateral incisor incurred in an RTA, with subgingival extension beyond junctional epithelium (Figures 2(a) and 2(b)). As the clinical scenario was similar to the first case, RCT and reattachment were planned in a similar manner.

After RCT and post space preparation (Figure 2(c)), an esthetic post (Glassix-NORDIN, Switzerland) of diameter 1.1 mm was selected.

To gain access to the gingival extent of the fracture line and to better evaluate its relation to the bone crest, buccal and palatal full thickness mucoperiosteal flaps were elevated using number 15 B-P blade. Hemostasis was achieved (Figure 2(d)). The post was luted and the fractured segment was reattached with resin cement (Multilink, Ivoclar, Vivadent). The flap was repositioned and sutured (Figure 2(e)), followed by physiological splinting for 2 weeks. At the 1-year followup, the tooth was clinically and radiographically healthy and the crown was esthetically satisfactory (Figure 2(f)).

### 2.3. Case  3

**Figure 3:**
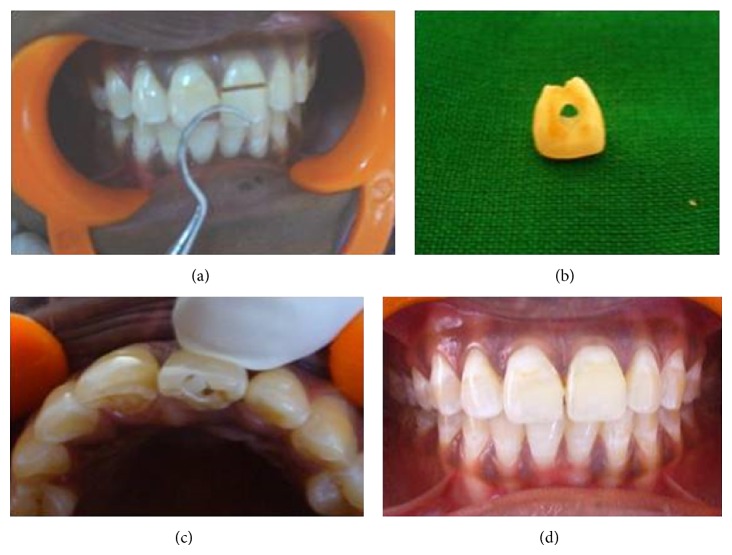
(a) Preoperative view. (b) Fractured segment. (c) Fragment reattached. (d) 1-year follow-up view.

The third case is of a 35-year-old female who presented with pain and mobility of her fractured left maxillary central incisor. Hard tissue examination revealed an Ellis class III fracture of 21 and a class I fracture of 11 (Figure 3(a)). RCT, fiber post (Glassix-NORDIN, Switzerland, diameter 1.1 mm) cementation, and reattachment of the fractured segment were planned and performed on 21 (Figures 3(b) and 3(c)). 11 was restored with composite resin. The 1-year follow-up clinical evaluation revealed acceptable aesthetics and function (Figure 3(d)).

## 3. Discussion

Conventional methods employed in the restoration of fractured teeth include partial and full coverage crowns, laminate veneers, and composite resins all of which are time-consuming, high priced, and not conservative [2]. First described by Chosack and Eidelman in 1964, restoration of fractured teeth using the dental fragment offers a fine way to reinstate the natural shape, contour, surface texture, occlusal alignment, and colour of the fragment [5]. In addition, tooth fragment reattachment allows restoration of the tooth with minimal sacrifice of the remaining tooth structure [3]. A growing number of case reports in the literature suggests that reattachment of a fractured tooth fragment is a viable approach for the treatment of coronal fracture of anterior teeth when the fractured segment is available [1–3]. The present paper reports 3 cases of successful reattachment of fractured segment of maxillary anterior teeth with 12-month followup.

The success of the reattachment depends on several factors: hydration of the fractured segment while outside oral cavity is one of them. This is necessary to maintain the vitality and original esthetic appearance of the tooth and also ensures adequate bond strength [2]. In all the reported cases, after the coronal segment was separated, hydration was ensured by storage in sterile isotonic saline.

When there is a substantial associated periodontal injury and/or invasion of the biological width, the restorative management of the coronal fracture should also consider the rehabilitation of those associated tissues [6]. In cases 1 and 3 reported in this paper, the fracture line extended subgingivally in the palatal area but not violating the biological width. In the second case too, the fractured line had a subgingival extension mesiopalatally. On clinical examination, it was seen that the biological width was only minimally invaded and hence, no osteotomy procedure was deemed necessary. Also, the restorative margin could be placed just above the level of the cementoenamel junction. To facilitate the perfect approximation of the fragments and finishing of the margins, full thickness mucoperiosteal flaps were elevated on both buccal and palatal aspects. The postsurgical healing phase remained uneventful.

Reinforcement of the reattached fragments using posts has been widely reported in the literature. Although many techniques with various materials have been suggested, resin-based restorative materials with tooth-colored fiber post may be considered the best option because of several advantages such as a suitable elastic modulus, esthetics, good bonding between post and cement, lower chair time, and minimal tissue removal [7, 8]. It is also reported that the use of a fiber post with fractured teeth, as it interlocks the two fragments, minimizes the stress on the reattached tooth fragment [7–9].

In addition to the preparation of the post space, in all cases a vent was created in the coronal separated segment as a leeway for the excess cement to flow out without buildup of any hydrostatic pressure. A similar technique has been recommended by Tosun et al. in reattachment using Ribbond material [10].

## 4. Conclusion

The 3 cases presented in this paper suggest that, with the materials available today along with appropriate clinical technique, reattachment of tooth fragment is a viable and conservative treatment option for fractured incisors. It is hoped that this report of 3 cases will add to the increasing volume of evidence which supports the viability of reattachment of the broken fragment of the anterior tooth reinforced by suitable restorations. Future reports may need to focus on reporting longer followup to bolster the evidence in favour of this treatment option.
